# Linkage of people experiencing homeless using two consent models.

**DOI:** 10.23889/ijpds.v7i3.1865

**Published:** 2022-08-25

**Authors:** Rob Trubey, Ian Thomas, Rebecca Cannings-John, Peter Mackie

**Affiliations:** 1 Centre for Trials Research, Cardiff University; 2 Administrative Data Research Centre Wales; 3 School of Geography and Planning, Cardiff University

## Objectives

Administrative data linkage is relatively under-utilised as a way of generating evidence to guide homelessness policy and service delivery in the UK. Our objective is to contribute insight into the ethical, legal, and practical challenges of using data linkage with data from people experiencing homelessness (PEH).

## Approach

We outline the data collection and linkage methodologies for two UK-based studies related to PEH. The first design aimed to explore the acceptability and feasibility of consented linkage of trial data (‘Moving On’ trial) to NHS Digital records in a cohort of recruited PEH in two English local authorities (n=50). The second design used administrative data originating from a local authority homelessness service in Wales (n=17,000 cases) to explore educational outcomes of children in homeless households. The resultant data linkage rates are contrasted and discussed in relation to the mechanisms for obtaining and linking personal data.

## Results

The Moving On trial demonstrated high rates of consent for data linkage and the ability to collect sufficient personal identifiable data to increase the chance of successful matching. Aggregate match rates will be discussed. Of the roughly 17,000 cases included in the local authority administrative data, 75% could be linked to unique individuals using probabilistic matching and were therefor ‘useable’ in linkage research. The proportion of useable cases rapidly decreased as the cut-off for matching quality was increased, to roughly 50% of cases being useable when a 99% match probability cut-off was used. Matching rates were higher amongst priority need homeless cases, possibly reflecting business need to identify and work closely with these people.

## Conclusion and Relevance

Where homelessness administrative data systems are not designed to enable data linkage, low matching rates can result, reducing study sample sizes and potentially leading to bias towards more extreme cases of homelessness if missed-matches are not random. Consented linkage within large-scale trials offers one possibility for generating long-term evidence.

